# Alcoholism Treatment in the United States: An Overview

**Published:** 1999

**Authors:** Richard K. Fuller, Susanne Hiller-Sturmhöfel

**Affiliations:** Richard K. Fuller, M.D., is director of the Division of Clinical and Prevention Research at the National Institute on Alcohol Abuse and Alcoholism, Bethesda, Maryland. Susanne Hiller-Sturmhöfel, Ph.D., is a science editor of Alcohol Research & Health

**Keywords:** addiction care, drug therapy, treatment research, United States, behavior therapy, cognitive therapy, Alcoholics Anonymous, motivational interviewing, treatment outcome, inpatient care, outpatient care, detoxification, aftercare, comparative study, patients, predictive factor, anti AOD (alcohol and other drug) craving agents, anti AOD abuse agents, intervention, literature review

## Abstract

On any given day, more than 700,000 people in the United States receive alcoholism treatment in either inpatient or outpatient settings. For many of those patients, detoxification—with or without pharmacotherapy—is the first step of treatment. The major behavioral approaches currently used in alcoholism treatment include cognitive-behavioral therapy, motivational enhancement therapy, and Alcoholics Anonymous (AA) or related 12-step programs. Clinical studies, such as the Project MATCH trial, have compared the effectiveness of these approaches. Overall, that study detected no significant differences among the three treatments in patient outcome, although certain treatment methodologies may be most appropriate for patients with certain characteristics. Pharmacotherapy with aversive or anticraving medications may supplement behavioral treatment approaches. Brief interventions that are delivered by primary health care providers also have been shown to reduce drinking levels, particularly in nondependent drinkers.

According to the 1992 National Longitudinal Alcohol Epidemiologic Survey, a national household survey, approximately 7.5 percent of the U.S. population (about 14 million Americans) abuse and/or are dependent on alcohol (Grant et al. 1994). Furthermore, according to the 1993 National Drug and Alcoholism Treatment Unit Survey, more than 700,000 people receive alcoholism treatment on any given day (National Institute on Alcohol Abuse and Alcoholism [NIAAA] 1997). Of those people, 13.5 percent receive treatment in either a residential or hospital (i.e., inpatient) setting, and 86.5 percent are treated on an outpatient basis. The approaches currently used in the treatment of alcohol problems generally have been developed based on three sources of information: (1) the experiences of recovering alcoholics and the professional staff treating them, (2) research on human behavior, and (3) studies of potential medications (i.e., pharmacological research).

Most treatment programs encourage patients to attend regular Alcoholics Anonymous (AA) meetings or similar self-help groups that are based on a 12-step philosophy. Many treatment programs also use relapse prevention techniques to help patients acquire the skills necessary to prevent a relapse after achieving initial abstinence. This approach is derived from therapeutic methods developed by behavioral psychologists. Cognitive-behavioral therapy (CBT) is based on learning theory principles, which posit that human behavior is largely learned and that learning processes can be used to change problem behaviors.

In addition to 12-step programs and behavioral therapies, one pharmacological agent, disulfiram (Antabuse^®^), has been available and used in alcoholism treatment since the late 1940s. In 1994 the U.S. Food and Drug Administration (FDA) also approved the medication naltrexone (ReVia^™^) for alcoholism treatment based on the results of randomized clinical trials. To date, however, naltrexone is not widely used, although pharmacotherapy has shown promising results in improving treatment outcome.

This article summarizes some of the characteristics and recent findings of alcoholism treatment research. It introduces the two general treatment settings (i.e., inpatient and outpatient) and reviews recent research on currently used alcoholism treatment approaches. These approaches include detoxification to manage alcohol withdrawal, non-pharmacological treatment methods, pharmacotherapy, and brief interventions that are designed to be delivered by primary care physicians rather than alcoholism treatment specialists. For more in-depth information on these topics, the reader is referred to subsequent articles in this issue.

## Basic Characteristics of Alcoholism Treatment Research

Until recently, few controlled clinical studies had evaluated and compared the efficacy of various treatment approaches, particularly of AA and other 12-step programs that are currently the cornerstone of alcoholism treatment in the United States. Several factors may contribute to the paucity of controlled research on the efficacy of AA. First, AA became a central component of most treatment programs before stringent study designs and criteria for assessing treatment outcome were introduced as standard procedures for determining alcoholism treatment efficacy. Second, researchers in the past have been deterred from studying AA for several reasons: AA programs can vary tremendously from group to group in the type and number of attendees as well as in the meeting style; furthermore, no standard definition of an AA member exists, and studying AA without perturbing its characteristics, such as the anonymity of its members, is difficult. Third, practitioners may be reluctant to enroll their patients in clinical studies of alcoholism treatments if the practitioners believe that the treatment to be evaluated is inferior to the traditional approaches already used. This reluctance is not limited to alcoholism treatment but also occurs in other treatment areas, such as breast cancer therapy. For example, when researchers first initiated clinical trials of lumpectomy (i.e., removal of only the tumor tissue rather than the entire breast) for breast cancer, many physicians considered it unethical to have their patients receive an operation that was potentially less effective.

In recent years, controlled and randomized clinical trials (i.e., studies in which patients are randomly assigned to different treatments) have become the standard for testing the efficacy of various therapies in alcoholism treatment research as well as in other areas of medical research. The practice of randomly assigning patients to different treatments is particularly important, because it minimizes the risk of obtaining biased results. For example, if patients with one characteristic (e.g., less severe dependence) are specifically assigned to one treatment and patients with another characteristic (e.g., more severe dependence) are specifically assigned to another treatment, researchers will not know whether any differences in efficacy between the treatments result from the differences in treatment, in patient characteristics, or both. If the patients are randomly assigned to the treatments, however, the patient characteristics in all treatment groups should be comparable, and differences in treatment efficacy will most likely result from differences in the treatments.

## Treatment Settings

Various alcoholism treatments differ not only in the methods they use but also in the setting in which they are delivered. Thus, alcoholism treatment can be performed either in residential and hospital (i.e., inpatient) settings or in outpatient settings. Inpatient rehabilitation programs traditionally last 28 days and provide highly structured treatment services, including group therapy, individual therapy, and alcoholism education. Furthermore, professional staff members are available around the clock to help manage the patient’s acute medical and psychological problems during the initial treatment period (i.e., detoxification). Alternatively, the patient may receive only short-term inpatient detoxification services before being transferred to an outpatient setting for further rehabilitation.

Currently, the vast majority of alcoholic patients are treated in outpatient facilities. Those programs offer alcoholism services of various intensity and duration. Day hospital programs (i.e., intensive outpatient programs) involve the patient for several hours per day, several days per week and were developed as alternatives to inpatient programs. Day hospital programs allow the patients to maintain their family roles while simultaneously receiving treatment. Less intensive out-patient services generally offer counseling sessions (i.e., group sessions, individual sessions, and—if necessary—family or couples therapy) once or twice per week. For many patients, those services are intended as maintenance therapy after the patients have received initial inpatient or intensive outpatient treatment.

Because of escalating health care costs, the focus in recent years has shifted away from inpatient treatment and toward outpatient treatment for all stages of recovery. This shift has resulted in an emphasis on outpatient detoxification and intensive outpatient services for initial treatment, approaches that are less expensive than inpatient treatment. In addition, the typical length of stay in inpatient programs has decreased substantially. The effectiveness of inpatient treatment versus outpatient treatment is controversial. Finney and colleagues (1996) concluded from their analysis of the findings of several studies that outpatient treatment is appropriate for most people with sufficient social resources and without co-occurring serious medical and/or psychiatric impairment. Conversely, inpatient treatment should be retained for clients with serious co-occurring medical and/or psychiatric conditions as well as for clients with few social resources and/or environments not supportive of recovery.

## Detoxification

Sudden cessation of alcohol consumption in people who have consumed alcohol regularly can lead to a variety of clinical symptoms that collectively are called alcohol withdrawal syndrome. The manifestations of alcohol withdrawal can range from mild irritability, insomnia, and tremors to potentially life-threatening medical complications, such as seizures, hallucinations, and delirium tremens. Consequently, before beginning long-term alcoholism treatment, many patients require a detoxification period during which they become alcohol free under controlled conditions. Depending on the severity of the withdrawal symptoms, those services can be delivered in either an inpatient or outpatient setting.

Medically supervised detoxification frequently involves treatment with medications (i.e., pharmacotherapy), particularly for patients with moderate to severe withdrawal symptoms. For most patients, benzodiazepines—a class of sedative medications that affect some of the same molecules in the brain as does alcohol—are the treatment of choice. An early randomized clinical trial demonstrated that benzodiazepines effectively prevented the development of delirium tremens (Kaim et al. 1969). Since that study was conducted, benzodiazepine use has revolutionized the treatment of alcohol withdrawal syndrome. Initially, benzodiazepines were administered on a predetermined dosing schedule for several days, often in gradually tapering doses. Recent studies have shown, however, that lower overall benzodiazepine doses can be used if the dosage is continually adjusted to the severity of the symptoms (Saitz 1998). Because benzodiazepines have an abuse potential of their own, therapists should not prescribe them after the acute withdrawal period.

Current state-of-the-art alcohol detoxification begins with an assessment of the severity of the patient’s withdrawal symptoms using such assessment tools as the revised Clinical Institute Withdrawal Assessment for Alcohol (CIWA–Ar) (Sullivan et al. 1989; Foy et al. 1988). This questionnaire evaluates the presence and severity of various withdrawal symptoms, such as nausea and vomiting; tremors; sweating; anxiety; agitation; tactile, auditory, and visual disturbances; headaches; and disorientation. The higher the patient’s score is on the CIWA–Ar, the greater is his or her risk for experiencing serious withdrawal symptoms, such as seizures and confusion.

Patients who experience only mild withdrawal symptoms according to the CIWA–Ar (i.e., score below 8 points) do not require pharmacotherapy; however, they should be monitored by their physician for potential complications. Conversely, patients who experience withdrawal symptoms that are either moderate (i.e., score from 8 to 15 points) or severe (i.e., score more than 15 points) should be treated with medications, such as benzodiazepines. Hayashida and colleagues (1989) demonstrated that patients with moderate withdrawal symptoms can be treated safely on an outpatient basis.

Hayashida (1998) has indicated that outpatient detoxification offers several advantages. For example, the patient may be able to use the same facility for both detoxification and subsequent long-term outpatient treatment. In addition, the patient may be able to more easily maintain family and social relationships and thus experience greater social support. Finally, the costs are lower for outpatient than for inpatient detoxification.

Outpatient detoxification is not appropriate, however, for patients who are at risk for life-threatening withdrawal symptoms, have other serious medical conditions, are suicidal or homicidal, live in disruptive family or job situations, or cannot travel daily to the treatment facility. Furthermore, outpatient detoxification is associated with significantly lower completion rates compared with inpatient detoxification (Hayashida et al. 1989). Finally, patients undergoing outpatient detoxification are at an increased risk of relapse during or shortly after detoxification because they have easier access to alcoholic beverages. However, long-term outcomes (i.e., more than 6 months) do not appear to differ between patients who receive inpatient or outpatient detoxification (Hayashida 1998).

## Behavioral Treatment Approaches and their Efficacy

The term “behavioral treatment” is used broadly here to include various nonpharmacological therapies whose objective is to change behavior (i.e., to reduce alcohol consumption). These approaches include behavioral therapy, cognitive therapy, various types of psychotherapy, counseling, and other rehabilitative strategies.

### Cognitive-Behavioral Therapy

One of the greatest challenges in the treatment of alcoholism and other addictions is the prevention of relapse. Patients have reported numerous factors that can trigger relapse. Some of those factors are internal to the patient, such as craving for alcohol, depression, and anxiety. Other factors are external, such as social pressure to drink; environmental cues associated with drinking (e.g., visits to bars or restaurants or the smell of alcohol); problems in relationships with other people; and negative life events, such as death or illness of a family member or loss of job. To prevent relapses resulting from those factors, CBT is designed to help the patient identify high-risk situations for relapse, learn and rehearse strategies for coping with those situations, and recognize and cope with craving. Variations of CBT are widely used in alcoholism treatment under the label of “relapse prevention.” In formal CBT, patients practice behavioral or cognitive skills to cope with high-risk situations through rehearsal, role playing, and homework.

Various studies have evaluated the efficacy of CBT (for more information on CBT, see the article in this issue by Longabaugh and Morgenstern, pp. 78–85). In the Project MATCH study, which compared the efficacy of three different treatment approaches, CBT achieved outcomes comparable to those of the other two therapies studied (Project MATCH Research Group 1997*a*). This result may be surprising, because CBT and other approaches, such as 12-step programs, appear to differ substantially. A recent review, however, identified common elements between 12-step programs and CBT-based approaches that may help explain their comparable results. For example, both approaches encourage the drinker to pursue activities incompatible with drinking and to identify and cope with negative thinking (McCrady 1994).

### Motivational Enhancement Therapy

Another psychological-behavioral approach to alcoholism treatment that is receiving increasing attention is motivational enhancement therapy (MET). This method, which is based on the principles of motivational psychology, does not guide the client step-by-step through recovery but strives to motivate the client to use his or her own resources to change his or her behavior. To that end, the therapist first assesses the type and severity of the patient’s drinking-associated problems. Based on this initial assessment, the therapist provides structured feedback to stimulate the patient’s motivation to change. The therapist also encourages the client to make future plans and, during subsequent counseling sessions, attempts to maintain or increase the client’s motivation to initiate or to continue implementing change. (For more information on MET, also see the article in this issue by DiClemente and colleagues, pp. 86–92.)

### AA and 12-Step Facilitation Therapy

AA and similar self-help groups outline 12 consecutive activities, or steps, that alcoholics should achieve during the recovery process. For example, these steps specify that drinkers must admit that they are powerless over alcohol, make a moral inventory of themselves, admit the nature of their wrongs, make a list of everyone they have harmed, and make amends to those people. Alcoholics can become involved with AA before entering professional treatment, as a part of their professional treatment, as aftercare following professional treatment, or instead of professional treatment. In addition, AA members can differ in the degree of their AA involvement (e.g., how often they attend AA meetings, whether they become involved with a sponsor, or whether they actively participate in meetings).

Twelve-Step Facilitation (TSF) is a formal treatment approach that has been developed to introduce clients to and involve them in AA and similar 12-step programs. Thus, TSF guides clients through the first five steps of the AA program and promotes AA affiliation and involvement. For example, therapists who use TSF actively encourage their clients to attend AA meetings, maintain a journal of their AA attendance and participation, obtain a sponsor, and work on completing the first five steps. In addition, the clients receive reading assignments from the AA literature.

Although AA is the most popular self-help group for people with drinking problems, its efficacy has rarely been assessed in randomized clinical trials. Most research on AA efficacy has compared the outcomes of people who did or did not become involved in AA. Those studies have reported a consistent association between voluntary AA participation and abstinence. Because the studies are not randomized, however, some factor other than AA involvement may account for abstinence. For example, possibly only people with certain characteristics (e.g., a greater motivation to become abstinent) choose to attend AA. Such potential differences between AA participants and nonparticipants may account for the treatment outcome. Consequently, one does not know whether the association between AA participation and abstinence is coincidental, results from client characteristics or similar factors, or is causally related.

To eliminate the possibility that another factor is responsible for the observed outcome and to demonstrate a cause-effect relationship between AA participation and outcome, researchers must conduct studies in which alcoholic patients are randomly assigned to AA and to one or more other treatments. To date, Walsh and colleagues (1991) and the Project MATCH Research Group (1997*a*) have conducted two major studies of the efficacy of either AA or involvement in AA using random patient assignment. The findings of the Project MATCH study are summarized in the following section.

The study by Walsh and colleagues (1991) included 227 alcohol-abusing participants whose employers had referred them to an employee assistance program (EAP). The participants were randomly assigned to one of three treatment options: (1) compulsory 3-week inpatient treatment followed by 1 year of attendance at AA meetings (i.e., hospital group), (2) compulsory attendance of AA meetings only (i.e., AA-only group), or (3) participants’ choice of treatment (i.e., choice group).^1^ The participants were followed for 2 years after their entry into the study. During that time, the investigators determined various drinking measures (e.g., abstinence rates), relapse rates (e.g., measured by the need for hospitalization for additional treatment), and work-related outcomes (e.g., proportion of participants who remained employed). The study results can be summarized as follows:

- On drinking measures, both the AA-only group and the choice group fared worse than the hospital group. For example, whereas 37 percent of the hospital group remained abstinent throughout the entire 2-year study period, only 17 percent of the choice group and 16 percent of the AA-only group were continuously abstinent. Similarly, the percentage of patients who did not become intoxicated during the study period was significantly higher in the hospital group than in either the choice or the AA-only group.
- The participants in the AA-only group relapsed more often than did participants in the other two groups. Thus, 63 percent of the AA-only group required hospitalization for a relapse during the 2-year study period, compared with 23 percent of the hospital group and 38 percent of the choice group. As a result of the additional treatment required by the AA-only group, the estimated total costs incurred by the hospital group were only an average of 10 percent higher than the costs incurred by the AA-only group.
- Work-related outcome variables, such as the proportion of patients who remained employed over the study period, did not differ significantly among the three groups.

This study is important for several reasons. First, the counselors involved in the study allowed their clients to be randomly assigned to a treatment. Second, the study methodology was scientifically sound, because it compared the outcomes of three treatment approaches to which the participants had been assigned randomly. Third, the results suggest that an approach which integrates AA with professional treatment generally will achieve better outcomes than will referral to AA alone. The study did not address, however, whether inpatient and outpatient professional treatments can be equally effective in combination with AA participation.

## Comparison of Different Treatment Approaches—Project Match

Project MATCH was a multisite study primarily focused on identifying patient characteristics that would predict which patients would benefit most from which treatment approach. The study included two groups of participants. One group (i.e., the aftercare sample) was recruited at four facilities that provided aftercare services to patients who had received inpatient or day-hospital treatment and therefore had received some kind of intensive treatment. The other group (i.e., the outpatient sample) was recruited at five outpatient facilities and comprised patients who had not received prior intensive inpatient or day-hospital treatment. As a result of their varied treatment histories, the two groups differed in certain patient characteristics. For example, the aftercare patients were more severely alcohol dependent when entering the study than were the outpatients.

Within both the aftercare and out-patient samples, participants were randomly assigned to receive either CBT, MET, or TSF. All interventions were delivered over a 12-week period in individual outpatient counseling sessions and were based on treatment manuals. To determine treatment efficacy, the study assessed several drinking-related variables. The primary variables, which were analyzed for the 90 days preceding treatment, the year following treatment, and the 90 days preceding the 3-year followup, were the percentage of days on which the participants were abstinent and the number of drinks consumed per drinking day.

**Table:** Overall Outcomes of Clients in the Aftercare and Outpatient* Groups of the Project MATCH Study

	Percentage of Clients Based on Treatment Group**	

Outcome Variable	Aftercare	Outpatient
Continuously abstinent for 1 year following treatment	35	20
Abstinent between 9 and 12 months after treatment	46	30
Drinking moderately without any problems between 9 and 12 months after treatment	7	12

* Aftercare clients were recruited into the study after receiving either inpatient or intensive outpatient treatment. Participants in the outpatient group received no intensive treatment before entering the study (Project MATCH 1997*a*).

** The numbers represent the proportion of clients in the aftercare and outpatient samples who fulfilled the outcome variable indicated. For example, 35 percent of all aftercare clients and 20 percent of all outpatient clients remained continuously abstient for 1 year following treatment.

### Outcome Differences Between Aftercare and Outpatient Samples

The study found that the aftercare sample generally achieved better treatment results than did the outpatient sample. For example, at 1-year followup, 35 percent of the aftercare patients had remained continuously abstinent, compared with 20 percent of the outpatient sample. Similarly, a higher percentage of the aftercare sample than of the out-patient sample was abstinent between 9 and 12 months after treatment or was drinking moderately without problems during that period (see table). Because the patients were not randomly assigned to either the aftercare or outpatient sample, however, one cannot conclude that aftercare is superior to outpatient treatment. Instead, a variety of factors may help explain why the aftercare patients more commonly achieved continuous abstinence. For example, the total amount of care received may contribute to treatment outcome, because the aftercare patients had received previous care in addition to the treatment approaches included in the study. Alternatively, the period of enforced abstinence that the aftercare patients experienced during their inpatient treatment may have had a beneficial effect.

### Outcome Differences Among Treatments

Although Project MATCH was not primarily concerned with comparing the three treatments for differential efficacy, the study’s design allowed such analyses because the participants were randomly assigned to the therapies.

In the aftercare sample, no differences were found in the efficacy of CBT, MET, and TSF during the year following treatment. Similarly, no differences or only small ones existed among the out-patients in the efficacy of the three treatments. Those differences that did exist usually indicated that TSF was most efficacious. For example, significantly more TSF-treated outpatients (i.e., 24 percent) than either MET- or CBT-treated outpatients (i.e., 14 and 15 percent, respectively) were continuously abstinent for 1 year after treatment (Project MATCH Research Group 1997*a*). Similarly, the abstinence rate during the preceding 90 days both at the 1- and 3-year followups was slightly higher among the TSF-treated outpatients than among the MET- and CBT-treated outpatients (Project MATCH Research Group 1998*a*).

Some differences existed in the time course in which the three treatments improved the outpatients’ drinking patterns; no such differences existed, however, among aftercare patients. Thus, during the 3 months of therapy, only 28 percent of MET-treated outpatients, compared with 41 percent of the CBT-and TSF-treated outpatients, were continuously abstinent or drank moderately without problems (Project MATCH Research Group 1998*b*). During the 3 years following treatment, however, the percentage of abstinent days and number of drinks per drinking day reported by the MET-treated outpatients were comparable with those of the CBT- and TSF-treated outpatients. These findings suggest that patients may achieve control over their drinking problems more slowly with the less directive MET approach than with the CBT or TSF approaches, but nevertheless experience long-term outcomes comparable with those of the two other therapies.

### Patient Characteristics Predicting Treatment Outcome

The primary goal of the Project MATCH study was to determine patient characteristics that could predict which treatment approach would be most effective for a given patient. The study identified four patient-treatment matches—one in the aftercare sample and three in the outpatient sample.

First, when the aftercare patients were classified according to the severity of their dependence, those patients who had been more severely dependent achieved better results (i.e., had more abstinent days and fewer drinks per drinking day) with TSF than with CBT (Project MATCH Research Group 1997*b*). For example, among the TSF-treated patients, the most severely dependent were abstinent on 94 percent of the days after treatment compared with abstinence on 84 percent of the days in the most severely dependent CBT-treated patients. Conversely, the least severely dependent CBT-treated patients averaged 94 percent of abstinent days after treatment, compared with 89 percent of abstinent days in the least severely dependent TSF-treated patients. These findings suggest that among patients who have already received inpatient treatment, TSF may be more appropriate for highly dependent patients, whereas CBT may be more appropriate for less severely dependent patients.

Second, in the outpatient sample, MET was the most effective approach in the treatment of patients with high levels of anger (as determined by the Spielberger Anger Scale). MET-treated outpatients with greater levels of anger had a greater percentage of abstinent days and fewer drinks per drinking day than did outpatients with similar anger levels who were treated with CBT. For example, MET patients with high anger levels were abstinent on 85 percent of the days compared with 75 percent of abstinent days for CBT patients with high anger levels (Project MATCH Research Group 1998*b*). This match between anger level and treatment approach was observed at the 1-year followup and persisted at the 3-year followup (Project MATCH Research Group 1998*a*).

Third, the Project MATCH results indicated that TSF and the resulting AA involvement was particularly effective for outpatients whose social networks (e.g., family members and friends) supported drinking. At the 3-year followup, those patients had better outcomes with TSF than with MET (Longabaugh et al. 1998). Thus, outpatients in the upper median^2^ for a supportive drinking network who received TSF had 83 percent of abstinent days, compared with 66 percent of abstinent days among similar patients receiving MET. AA involvement was an important mediator of this effect: TSF-treated patients whose social network supported drinking and who became involved in AA had 91 percent of abstinent days compared with 60 percent of abstinent days for similar patients who did not become involved in AA. AA involvement also enhanced treatment outcome in patients whose social networks were supportive of drinking and who received either MET or CBT; however, this beneficial effect of AA involvement was smaller than among patients receiving TSF.

Researchers also observed the relationship among a drinker’s social network, AA involvement, and treatment outcome in a recent long-term study of patients at 15 Department of Veterans Affairs’ hospitals^3^ (Humphreys et al. in press). The study found that replacing patients’ social networks of drinking friends with the AA fellowship was at least in part responsible for the better outcomes observed in clients who became involved with AA. Thus, treatment approaches that facilitate the clients’ involvement in 12-step programs may be beneficial, particularly for clients whose social networks support drinking. For those people, a new social network of friends who support abstinence appears to be a key element in recovery.

Fourth, the Project MATCH findings indicated that for the first 9 months following treatment, outpatients who were low in psychiatric severity as assessed by the Addiction Severity Index psychiatric subscale experienced more abstinent days and fewer drinks per drinking day when treated with TSF than with CBT. At the 1-year followup, however, this difference between the treatment groups no longer existed.

Overall, the results of Project MATCH provide only limited support for the hypothesis that patients can be matched with optimal treatments based on patient characteristics, because only 4 out of a possible 21 matches (based on the number of treatments and patient characteristics evaluated) were detected. Furthermore, one of those four matches had dissipated within 1 year after treatment. The findings suggest, however, that some incremental improvement in outcome occurs if aftercare patients are screened for severity of dependence and outpatients are screened for anger and type of social network prior to treatment.

## Pharmacotherapy

Currently, therapists primarily use two types of medications in alcoholism treatment: (1) aversive medications, which deter the patient from drinking, and (2) anticraving medications, which reduce the patient’s desire to drink.

### Aversive Medications

The most commonly used aversive medication in alcoholism treatment is disulfiram, which has been available since the late 1940s. The medication causes an unpleasant reaction (i.e., nausea, vomiting, flushing, and increased blood pressure and heart rate) when the patient ingests alcohol. Early clinical studies of disulfiram therapy reported favorable outcomes (i.e., improved abstinence rates) among recovering alcoholics; however, most of those studies were not conducted according to the current standards of controlled clinical trials (Fuller and Roth 1979).

Conversely, according to one large, well-designed study, disulfiram did not increase the rate of sustained abstinence or time to relapse among the patients (Fuller et al. 1986). In addition, only a subgroup of study participants (i.e., patients who showed evidence of greater social stability) drank less frequently when taking disulfiram than did patients with similar characteristics who received an inactive medication (i.e., a placebo) or no medication. Furthermore, abstinence was related to the patients’ compliance with the medication regimen (i.e., whether the patients continued to take the medication regularly). Because poor compliance can nullify disulfiram’s effectiveness, some programs require staff members or relatives to observe the patient ingesting the medication. A randomized study (Chick at al. 1992) found that supervised disulfiram administration was more beneficial than supervised vitamin administration.

### Anticraving Medications

Various brain chemicals have been implicated in mediating alcohol’s pleasant effects and in contributing to the development of tolerance to and craving for alcohol. Accordingly, researchers have attempted to prevent alcohol’s pleasant effects and craving for alcohol by developing medications that interfere with the actions of those brain chemicals. Two of those medications are naltrex-one and acamprosate.

Naltrexone was the first agent in nearly 50 years to be approved by the FDA for alcoholism treatment. The approval was based on two randomized clinical trials reporting that naltrexone combined with psychosocial treatment reduced 3-month relapse rates from 50 percent among patients who received a placebo to 25 percent among patients who received naltrexone (O’Malley et al. 1992; Volpicelli et al. 1992). As with disulfiram, a recent study found that compliance with naltrexone was critical for obtaining favorable outcomes (Volpicelli et al. 1997). Naltrexone acts by interfering with the actions of key brain chemicals called endogenous opioids. In response to alcohol, endogenous opioids activate certain brain cells and induce some of alcohol’s pleasant effects (e.g., euphoria and reduced anxiety). By blocking the actions of endogenous opioids, naltrexone prevents alcohol from exerting its pleasant effects and may reduce the patient’s desire to drink.

Acamprosate is another medication aimed at reducing alcohol craving. Researchers in Europe have studied the drug extensively; however, it is not yet commercially available in the United States. Scientists still do not know acamprosate’s precise mechanism of action. However, the drug appears to interact with a certain type of receptor (i.e., the *N*-methyl-d-aspartate [NMDA] receptor) that is located on the surface of some brain cells and mediates the effects of another important brain chemical, glutamate. Controlled European studies have found that acamprosate treatment can almost double the abstinence rate among recovering alcoholics (Sass et al. 1996). Researchers in the United States are currently conducting a multisite randomized clinical trial of acamprosate.

### Further Directions in Pharmacotherapy

In addition to the medications described here, scientists are evaluating other pharmacotherapeutic approaches to alcoholism treatment (for more information on recent advances and future trends in pharmacotherapy, see the article in this issue by Johnson and Ait-Daoud, pp. 99–106). For example, some researchers are testing medications targeting other brain chemicals (e.g., serotonin) that have been implicated in mediating alcohol’s effects. To date, however, clinical trials of serotonin-targeting agents have not demonstrated efficacy in alcohol-dependent patients (Kranzler et al. 1995; Johnson et al. 1996).

Some alcoholics suffer from co-occurring psychiatric conditions, such as depression and anxiety. In some patients, these psychiatric conditions precede, and possibly even precipitate, alcohol abuse and dependence. In other patients, the psychiatric condition results from long-term alcohol abuse. It is plausible that at least in the former group of patients, treatment of the psychiatric illness may decrease alcohol consumption, because the patients no longer need to resort to alcohol to alleviate anxiety or depression. Three clinical trials of antidepressant medication therapy for alcoholism found that this treatment improved the patients’ depression (Mason et al. 1996; McGrath et al. 1996; Cornelius et al. 1997). However, only one of those studies found that antidepressant therapy caused a major change in drinking levels (Cornelius et al. 1997). Studies of the anti-anxiety medication buspirone in alcoholic patients have yielded conflicting results (Kranzler et al. 1994; Malcolm et al. 1992).

Finally, other clinical trials are evaluating whether treatment efficacy can be increased by combining medications, because combination therapy is effective for the treatment of many other conditions, such as high blood pressure. Researchers and clinicians hope that these approaches will yield effective therapies to help alcoholics achieve long-term abstinence.

## Brief Interventions

Many people with alcohol-related problems do not seek the help of an alcoholism treatment specialist but receive their care from a primary care provider. Usually conducted in a primary care setting, brief intervention treatments last for up to four or five office visits. In general, such interventions begin with an assessment of the extent of the patient’s alcohol-related problems (e.g., impaired liver function or alcohol-related problems at work) and a discussion of the potential health consequences of continued drinking. The health care professional then offers advice on strategies to either cut down on drinking (for non-alcohol-dependent patients only) or abstain from drinking (for both dependent and nondependent patients). Such strategies can include setting specific goals for reducing the number of drinks consumed per day or per week and agreeing to written contracts that specify measures of progress toward changes in drinking behavior (for more information on such contracts, see the article in this issue by Higgins and Petry, pp. 122–127).

Two controlled studies conducted in the United States and Canada have investigated the efficacy of brief interventions. Those studies demonstrated that brief interventions reduced drinking (Fleming et al. 1997; Israel et al. 1996), alcohol-related problems (Israel et al. 1996), and the patient’s use of health care services (Fleming et al. 1997). The current challenge is to educate health care professionals about and motivate them to use brief interventions (for more information on brief interventions, see the article in this issue by Fleming and Manwell, pp. 128–137).

## Conclusions

The past decade has seen remarkable advances in alcoholism treatment research. Researchers and treatment providers now have a better understanding of the effectiveness of nonpharmacological treatments and of key elements in 12-step programs. In addition, research on effective pharmacotherapies for alcoholism is entering a new era. Finally, brief interventions delivered in primary care settings have been shown to be effective in reducing drinking among people who have alcohol-related problems or who are at risk for such problems.

Substantial challenges remain, however, before the results of this research can be translated into improved treatment outcomes. For example, many treatment programs do not use pharmacotherapies, primarily for philosophical reasons—that is, treatment providers are reluctant to substitute one drug (i.e., the treatment medication) for another (i.e., alcohol). Similarly, many primary care providers may not be aware of the usefulness and correct use of brief interventions. Consequently, all health care professionals working with people who abuse or are dependent on alcohol—particularly addiction professionals—must stay informed about improvements in alcoholism treatment and novel treatment options. Otherwise, patients with alcohol-related problems who might benefit from new approaches, such as pharmacotherapies, might be deprived of an opportunity for achieving long-term recovery.

## Footnotes

1 Although allowing patients to choose their treatment is not a standard treatment approach, the researchers included such a group, because some treatment researchers thought that involving patients in planning their treatment would improve outcome.

2 The upper median is the 50 percent of people in a sample who have the highest scores on a given variable (e.g., on an index of a network supportive of drinking).

3 The patients in that study had not been assigned randomly to a specific treatment approach.
